# From structure to function, how bioinformatics help to reveal functions of our genomes

**DOI:** 10.1186/s13059-017-1320-1

**Published:** 2017-09-26

**Authors:** Xiaoqi Zheng, Sheng Zhong

**Affiliations:** 1 Department of Mathematics, Shanghai Normal University, Shanghai, 200234 China; 2 Department of Bioengineering, UC San Diego, San Diego, California 92093 USA; 3 Institute for Genome Medicine, UC San Diego, San Diego, California 92093 USA

**Keywords:** Bioinformatics, Non-coding RNAs, RNA editing, Chromosome structure, Transposable elements, Cancer genomes

## Abstract

A report on the 13th International Bioinformatics Workshop held in Harbin, China, 5–6 August 2017.

## Introduction

The International Bioinformatics Workshop (IBW), held every other year in China since 2003, has grown into an international forum for showcasing the most important breakthroughs in bioinformatics-related fields. At this year’s IBW, the topics of several presentations were at the center of attention, including novel functional features of genomes and transcriptomes, three-dimensional genome organization, and recent evolutions of mammalian and human genomes. Here, we summarize a subset of IBW’s presentations on these topics.

## Mining genomic dark matter

Approximately 30% of the human genome comprises repetitive sequences known as transposable elements (TEs). Functional analyses of the human genome have paid little attention to TEs; however, the revelation that previously so-called “junk” DNA might be functional has aroused intrigue in this “dark matter” of the genome.

By showing the birth of new exons derived from the insertion of *Alu* elements, Yi Xing (University of California, Los Angeles, USA) revealed one novel function of genomic dark matter. *Alu*-derived exons exhibit diverse alternative splicing patterns in various human tissues. As well as their role in the modulation of translation efficiency, some of these *Alu*-originated exons also encode new peptides. How do TEs shape human transcriptional networks? Ting Wang (Washington University in St. Louis, USA) gave the example of AluJb, a subfamily of *Alu* sequences, which, when inserted into the genome, gave rise to alternative promoters to activate oncogene LIN28B. Knocking out a specific copy of AluJb in a lung cancer cell line suppressed cell growth and migration.

## Cryptic functions of RNA shortening and synonymous mutation

Wei Li (Baylor College of Medicine, USA) reported a novel mechanism of 3′ UTR shortening that leads to the repression of tumor suppressor genes by disrupting crosstalk with competing endogenous RNA. At least in part, this process is mediated by an RNA cleavage factor CFIm25, which may be responsible for regulating alternative polyadenylation sites on thousands of messenger RNAs.

Synonymous single-nucleotide variants (sSNVs) are usually left out of analyses because of the absence of resulting amino acid changes. However, by using regSNPs-splicing software to prioritize sSNVs associated with RNA splicing, Yunlong Liu (Indiana University, USA) found that disease-causing sSNVs are enriched in protein functional domains. Potential functional enrichment of intronic single-nucleotide variants was also discussed.

## Tools to identify novel functional features of transcriptomes

Yi Xing (University of California, Los Angeles, USA) presented rMATS-turbo, an updated version of rMATS that allows ultra-fast detection of differential alternative splicing and isoforms from replicated RNA-seq data. Shirley Liu (Harvard University, USA) described TRUST (https://bitbucket.org/liulab/trust/), a tool to assemble T-cell receptor hypervariable region sequences by assigning informative unmapped reads from tumor RNA-seq data into T-cell receptor genes. Wei Wang (University of California San Diego, USA) proposed the Taiji pipeline (https://github.com/kaizhang/Taiji) to construct gene regulatory networks and identify key regulators of a specific cell stage by integrating multi-type high-throughput sequencing data including RNA-seq, open chromatin, and histone modifications.

## Circular RNA, RNA editing, and interactions

Circular RNA (circRNA) is endogenous non-coding RNA with covalently linked 3′ and 5′ ends that form a backsplicing structure. It is tissue-specific and evolutionarily conserved, suggesting a potential functional role. Fangqing Zhao (Beijing Institutes of Life Science, Chinese Academy of Sciences [CAS], China) reported the prevalence of alternative splicing within circRNAs and their tissue-specific expression patterns. To help explore this line of work, the Zhao group developed a new method based on the backsplicing and reverse overlap features of circRNA. This method was able to recover ~ 80% full transcripts of circRNAs in cells (unpublished data). Li Yang (CAS–Max Planck Gesellschaft [MPG] Partner Institute for Computational Biology, China) discussed the species-specific expression of circRNAs from an evolutionary perspective. He reported that the rapidly evolved SINEs (short interspersed nuclear repetitive DNA elements), especially *Alu* elements in humans, are involved in the biogenesis of circRNAs.

RNA editing post-transcriptionally alters RNA sequences by substituting, deleting, or inserting nucleotides that can cause changes in RNA structure or the resulting protein product(s). Han Liang (The University of Texas MD Anderson Cancer Center, USA) reported the effect of A-to-I RNA editing on microRNAs (miRNAs) and its function in cancer. Liang and colleagues identified miR-200b through a pan-cancer analysis of TCGA transcriptomic data. The level of editing of this microRNA revealed distinct patterns associated with patient survival time compared with the primary miRNA. Specifically, unedited miR-200b inhibited epithelial–mesenchymal transition and suppressed tumor metastasis. In contrast, with only a single nucleotide modification in the mature region, the edited miR-200b was able to promote the migration and invasion of cancer cells by retargeting a new set of genes that included a key metastatic suppressor, LIFR. This striking example highlights the importance of RNA editing in cancer development.

Determining the interactions between different types of RNA, or between RNA and chromatin, is key to understanding their functions. Sheng Zhong (University of California, San Diego, USA) reported two techniques, MARIO and MARGI, for the massive detection of RNA–RNA and RNA–chromatin interactions in vivo. Zhong also introduced the plans of the 4D Nucleome consortium (https://www.4dnucleome.org/) for revealing genome architecture and nuclear organization.

## Visualization and analysis of genomic interaction data

New methods and tools have been developed to analyze and present genomic interaction datasets. Yun Li (University of North Carolina, Chapel Hill, USA) described HUGIn (http://yunliweb.its.unc.edu/HUGIn/), a unified web browser for visualizing and annotating Hi-C data from human primary tissues and cell lines. Zhihua Zhang (Beijing Institute of Genomics, CAS, China) described Delta, a new 3D genome visualization tool, and DeDoc, a new method for calling topologically associated domains by merging and combining structural coding trees. Notably, DeDoc can stably detect topologically associated domains with just a few pieces of single cell Hi-C data.

Another common task required in the analysis of genomic interaction data is to identify long-range genomic interactions. Jian Ma (Carnegie Mellon University, USA) presented PEP, a tool to predict enhancer–promoter interactions using only sequence-based features.

## Genome evolution, adaptation, and personal variations

Wenfeng Qian (Institute of Genetics and Developmental Biology, CAS, China) discussed the relationship between genetic interactions (epistasis) and the order of eukaryotic genes on a chromosome. By extending theories from population genetics, Qian proposed the hypothesis that genetic interaction networks might drive the evolution of gene order. In support of his hypothesis, analysis of the global genetic interaction network recently published in budding yeast indeed revealed an anti-correlation between epistasis and gene distance. This was partially attributed to genes exhibiting positive epistasis having a tendency to translocate close to each other on a chromosome during evolution.

Two of the speakers described genome-wide association studies to investigate the genetic basis of high altitude adaptation in humans and the Tibetan mastiff dog from the Tibet Plateau. Shuhua Xu (CAS–MPG Partner Institute for Computational Biology, China) investigated the genetic origin of high altitude adaption in Tibetans using deep-sequenced whole genome data. Using ArchaicSeeker, a tool developed by his own group, he suggested that the Tibetan people are an admixture of multiple populations, with an ancestry derived from both archaic and modern human groups. Xu also proposed a “fitness-borrow” hypothesis to explain the mechanism of altitude adaptation in Tibetans and Sherpas. Yixue Li (Shanghai Institutes for Biological Sciences, CAS, China) investigated the genetic basis of hypoxia adaptation in the Tibetan mastiff. He identified two loci for the genes *EPAS1* and *HBB* that are associated with hypoxia tolerance, and suggested that this trait originated from Tibetan gray wolves.

Ge Gao (Peking University, China) reported on COPE, a genomic variant annotation tool that accounts for the cumulative effects of multiple variants within the same loci. COPE identifies multiple function-changing variants that are neglected by conventional tools from the 1000 Genomes dataset. Kai Ye (Xi’an Jiaotong University, China) presented Pindel-C (https://github.com/genome/pindel), a tool for detecting complex indels and structural variations from next-generation sequencing data. Pindel-C detected complex indels in 285 cancer genes from The Cancer Genome Atlas, which were missed in previous studies. Interestingly, applying Pindel-C to whole genome sequencing data of 250 trio-families from the Genome of the Netherlands project, Ye also found that most germline mutations are of paternal origin.

## Conclusions

As commented by Xiaole Shirley Liu (Harvard University, USA), bioinformatics emerged as an auxiliary tool in biomedical research and has grown into an independent discipline at the forefront of biological discovery and applications. IBW participants have become increasingly diverse over the years, perhaps reflecting the increasing collaboration between computational and experimental biologists as well as biomedical practitioners.

## Abbreviations

CAS: Chinese Academy of Sciences
ceRNA: Competing endogenous RNA
circRNA: Circular RNA
Hi-C: High-throughput chromosome conformation capture
IBW: International Bioinformatics Workshop
miRNA: MicroRNA
sSNV: Synonymous single-nucleotide variant
TAD: Topologically associated domain
TE: Transposable element

